# Supplementary material for: A fault-intrusion-tolerant system and deadline-aware algorithm for scheduling scientific workflow in the cloud
Source: PeerJ Comput Sci. 2021 Nov 2;7:e747. doi: 10.7717/peerj-cs.747 (PMC8576554; doi:10.7717/peerj-cs.747)
Supplement: Supplemental Information 6 [file peerj-cs-07-747-s006.zip › Myproject1.1/docs/org/cloudbus/cloudsim/package-tree.html]

org.cloudbus.cloudsim Class Hierarchy


JavaScript is disabled on your browser.


- Overview
- Package
- Class
- Use
- Tree
- Deprecated
- Index
- Help

- Prev
- Next

- Frames
- No Frames

- All Classes

# Hierarchy For Package org.cloudbus.cloudsim

Package Hierarchies:

- All Packages

## Class Hierarchy

- java.lang.Object
  - org.cloudbus.cloudsim.Cloudlet
  - org.cloudbus.cloudsim.CloudletScheduler
    - org.cloudbus.cloudsim.CloudletSchedulerSpaceShared
    - org.cloudbus.cloudsim.CloudletSchedulerTimeShared
      - org.cloudbus.cloudsim.CloudletSchedulerDynamicWorkload
  - org.cloudbus.cloudsim.Consts
  - org.cloudbus.cloudsim.DatacenterCharacteristics
  - org.cloudbus.cloudsim.DataCloudTags
  - org.cloudbus.cloudsim.File
  - org.cloudbus.cloudsim.FileAttribute
  - org.cloudbus.cloudsim.HarddriveStorage (implements org.cloudbus.cloudsim.Storage)
    - org.cloudbus.cloudsim.SanStorage
  - org.cloudbus.cloudsim.Host
    - org.cloudbus.cloudsim.HostDynamicWorkload
  - org.cloudbus.cloudsim.HostStateHistoryEntry
  - org.cloudbus.cloudsim.InfoPacket (implements org.cloudbus.cloudsim.Packet)
  - org.cloudbus.cloudsim.Log
  - org.cloudbus.cloudsim.NetworkTopology
  - org.cloudbus.cloudsim.Pe
  - org.cloudbus.cloudsim.ResCloudlet
  - org.cloudbus.cloudsim.core.SimEntity (implements java.lang.Cloneable)
    - org.cloudbus.cloudsim.Datacenter
    - org.cloudbus.cloudsim.DatacenterBroker
  - java.lang.Throwable (implements java.io.Serializable)
    - java.lang.Exception
      - org.cloudbus.cloudsim.ParameterException
  - org.cloudbus.cloudsim.UtilizationModelFull (implements org.cloudbus.cloudsim.UtilizationModel)
  - org.cloudbus.cloudsim.UtilizationModelNull (implements org.cloudbus.cloudsim.UtilizationModel)
  - org.cloudbus.cloudsim.UtilizationModelPlanetLabInMemory (implements org.cloudbus.cloudsim.UtilizationModel)
  - org.cloudbus.cloudsim.UtilizationModelStochastic (implements org.cloudbus.cloudsim.UtilizationModel)
  - org.cloudbus.cloudsim.Vm
  - org.cloudbus.cloudsim.VmAllocationPolicy
    - org.cloudbus.cloudsim.VmAllocationPolicySimple
  - org.cloudbus.cloudsim.VmScheduler
    - org.cloudbus.cloudsim.VmSchedulerSpaceShared
    - org.cloudbus.cloudsim.VmSchedulerTimeShared
      - org.cloudbus.cloudsim.VmSchedulerTimeSharedOverSubscription
  - org.cloudbus.cloudsim.VmStateHistoryEntry

## Interface Hierarchy

- org.cloudbus.cloudsim.Packet
- org.cloudbus.cloudsim.Storage
- org.cloudbus.cloudsim.UtilizationModel

- Overview
- Package
- Class
- Use
- Tree
- Deprecated
- Index
- Help

- Prev
- Next

- Frames
- No Frames

- All Classes
